# Recover From Failure: Examining the Impact of Service Recovery Stages on Relationship Marketing Strategies

**DOI:** 10.3389/fpsyg.2022.852306

**Published:** 2022-04-04

**Authors:** Jie Gao, Lixia Yao, Xiao Xiao, Peizhe Li

**Affiliations:** ^1^Lucas College and Graduate School of Business, San José State University, San Jose, CA, United States; ^2^School of Business Administration, Zhejiang Gongshang University, Hangzhou, China; ^3^School of Community Resources and Development, Arizona State University, Phoenix, AZ, United States

**Keywords:** service failure, consumer loyalty, relationship quality, digital transformation, restaurant, small business, compensation

## Abstract

**Purpose:**

Given the digital transformation of service businesses by providing online food services and the influence of online reviews on consumers’ purchasing decisions, this study examines how service recovery attributes in different stages influence relationship marketing strategies, i.e., relationship quality and customer loyalty after service failure. This study is built upon a revised service recovery cycle model by accounting for three stages and their corresponding attributes; whereon a conceptual stage model of service recovery is proposed. This conceptual stage model incorporates stages of service recovery, their respective attributes, and how they influence relationship marketing strategies.

**Design/methodology/approach:**

An online marketing company was employed for data collection in 2019, which resulted in 301 valid responses. A Structural Equation Model (SEM) was conducted with all the data to test the relationships between the constructs. The individual measurement model was tested using the Exploratory Factor Analysis (EFA) and Confirmatory Factor Analysis (CFA). A structural model was estimated using AMOS to test all the hypotheses.

**Findings:**

The findings demonstrate that the attributes (i.e., response speed, compensation) paired with the first two stages of service recovery can significantly influence consumer loyalty in a positive state. The findings also manifest the intermediary role that relationship quality has played in the association of service recovery and consumer loyalty, which implies that the food delivery businesses could attain a more comprehended relationship quality with consumers through active and timely compensatory service recovery consumer loyalty to the food businesses.

**Originality/value:**

This study examines how these different stages of the service recovery cycle influence the decision-making of relationship marketing strategies (i.e., relationship quality, customer loyalty) on the prerequisite of service failure. This study aspires to expand the service recovery research by objectifying a conceptual stage model of service recovery, incorporating stages’ recovery attributes and how these recovery attributes reciprocally influence relationship quality and customer loyalty.

## Introduction

As part of business strategies tackling the challenge of COVID, more service businesses have sped up their real-life, real-time digital transformation, including online food services, meal delivery solutions, etc. In the transformation, loyal customers have been important for businesses to maintain growth and profitability; thus, business owners and executives consistently strive to develop and maintain positive customer relationships (Reichheld, 1996). In the service industry, however, it is impossible to receive perfect reviews. Thus, the post-service follow-up and recovery has become an essential job (Bilgihan et al., 2014). When customers encounter service failure, their dissatisfaction may lead to negative reviews that reduce their intention to return and future consumption (Alexandrov et al., 2013; Aguilar-Rojas, 2015). A majority of consumers have indicated a higher level of trust on online reviews (Nissen, 2012). They would particularly read more negative reviews that have a greater impact on their decision-making process (Zhang et al., 2018).

In the online service context, service problems and failures occur more often, compared with traditional service contexts (Harris et al., 2006). Given the fact that consumers are very likely to post their reviews and comments on review platforms as public references, businesses necessitate in following up with their customers and conducting service remediation if needed. A responsive business would take commitment and time, responding to positive customer interactions, and negative reviews and comments for multiple purposes of customer happiness and retention, page engagement on social media and online reputation management. Service recovery has been imperative for business to maintain positive customer relationships and reconstruct customer loyalty in the context of online service failure (Sparks et al., 2016). Existing evidence has primarily focused on the consequences of service failure; however, limited studies has ever looked at how to respond to online service failure by accounting for recovery stages and attributes (Di Pietro, 2012).

Given the challenges that business owners have in the online service context to turn unhappy customers into loyal fans, this study is built upon a revised service recovery cycle model by accounting for three stages and their corresponding attributes, and examines how service recovery attributes in different stages influence relationship marketing strategies, i.e., relationship quality and customer loyalty after service failure. This study aspires to expand the service recovery literature by proposing a conceptual stage model of service recovery, incorporating recovery attributes by stages, and understanding how they reciprocally influence relationship quality and customer loyalty.

## Literature Review

### Service Recovery Stages and Their Attributes

The concept of service recovery was first proposed by Etzel and Silverman (1981) and refers to the measures taken by service providers to compensate for the loss of consumers after the occurrence of service errors in response to negative evaluations by the consumers to change consumers’ attitudes toward enterprises (Groonros, 1988). Service recovery is a management process. Companies need to know that service failures have occurred, identify the reasons for such losses, evaluate their impact, and carry out appropriate management activities to resolve these failures (Tax et al., 1998). Based on this perspective, service recovery is defined as the actions that organizations take in response to a service failure (Steyn et al., 2011) or the process of addressing mistakes (Hu et al., 2013). Implementing effective service recovery after service failures does not necessarily lead to negative results (Hu et al., 2013). Particularly in the restaurant business, service failure is difficult to avoid (Namkung and Jang, 2010; Byun and Jang, 2019), emphasizing the importance of practical service recovery efforts in a restaurant setting. Service failures are effectively managed in a restaurant setting also affects customers’ behavioral intentions, including word-of-mouth (WOM) intention and revisit intention (Ok et al., 2006). During the recovery process, customers usually expect fairness to be involved in compensation for the loss during the service failure. Many researchers have indicated that organizations can use several strategies to recover from service failures, including communicating with customers to provide feedback, offering explanations for their losses (Boshoff and Staude, 2003; La Khanh, 2004). They also apologize for their losses (Mattila and Cranage, 2005; Mostert et al., 2009). Thus, service failures have a positive relationship with service recovery.

**FIGURE 1:**
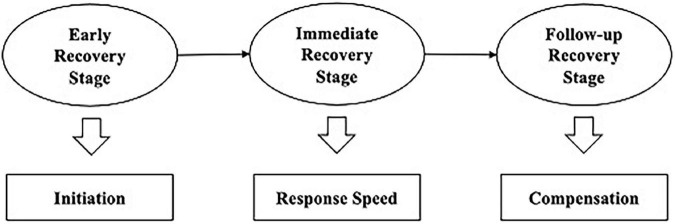
The revised service recovery cycle model.

This manuscript provides insight into the dynamics of relationship repair by accounting for the impact of time-specific factors on the relationship repair process. Thus, we propose the revised service recovery cycle model (Figure 1), which includes the following three primary stages of service recovery and their attributes: (1) initiation at the early recovery stage; (2) response speed at the immediate recovery stage; and (3) compensation at the follow-up recovery stage.

First, service recovery encompasses a much broader set of activities than addressing complaints because it includes situations in which a service failure occurs the customer lodges but no complaint. Evidence shows that a majority of dissatisfied customers do not bother to complain (Harari, 1992). Prior research has focused solely on failure/recovery situations in which customers have filed a formal complaint with an organization (Tax et al., 1998). Several researchers have suggested that proactive recovery efforts enhance customers’ evaluations of the service provider (Berry, 1995). When an organization initiates recovery during the early stage, the customer is likely to view the proactive effort as an act of courtesy, a demonstration of honesty and forthrightness, and a show of empathic understanding and respect.

Second, we argue that recovery speed is a critical factor during the immediate recovery stage. Recovery speed refers to service providers’ promptness in responding to service failure, which influences consumer satisfaction (Crisafulli and Singh, 2017). Evidence suggests that longer recovery time would lead to additional negative consequences, such as negative WOM and lower levels of recovery satisfaction (Hogreve et al., 2017; Babin et al., 2021). Service providers can reduce these negative impacts by quickly responding to consumers’ complaints and/or negative reviews, and conducting service remediation.

Lastly, consumers perceive inequity following a service failure, and they might be appeased when provided with suitable compensation during the follow-up stage. According to social exchange theory, compensation (e.g., discounts, free merchandise, refunds, and coupons) by an organization could balance the costs and benefits of achieving an equitable exchange in relationship recovery. Walster et al. (1973) showed that compensation is used to restore equity in an exchange relationship when another party has harmed one party. Tax et al. (1998) performed a content analysis of qualitative evaluations of service complaint experiences and showed that compensation is the most essential recovery dimension. Therefore, compensation plays a vital role during the follow-up recovery stage after service failures.

It is essential to differentiate between service recovery and service failure. Service failure is defined as a “situation where a service provider does not meet customer expectations in terms of its service products or engages in service behaviors that customers evaluate as unsatisfactory” (Harrison-Walker, 2012). Service failures can be classified into the following three types: (1) core service failures, such as failures to fulfill basic service needs (Yang and Mattila, 2012); (2) interactional service failures, including the attitudes and behaviors of employees during face-to-face interactions with customers, such as a server treating a customer impassively or impolitely (Yang and Mattila, 2012); and (3) process service failures, which involve how the core service is delivered to the customer, such as slow service or incorrect delivery order (Mohr and Bitner, 1995). Service failure can result in dissatisfied customers and negative WOM. Thus, recovery efforts are critically needed in service failure situations, and service recovery is among the critical antecedents of customer satisfaction and loyalty (Craighead et al., 2004).

The service recovery paradox (SRP) has emerged in the marketing literature as an essential effect of service failures. The SRP is defined as a situation in which post-recovery satisfaction is greater than the satisfaction before the service failure when customers experience high recovery performance (Smith and Bolton, 1998; Maxham, 2001). Effective service recovery may lead to a higher level of satisfaction than the periods when the service was incorrectly performed; recovery encounters offer an opportunity for service providers to increase customer retention (Hart et al., 1990). Based on the disconfirmation framework (McCollough et al., 2000; Oliver, 2010), the SRP is related to secondary satisfaction following a service failure in which customers compare their expectations for recovery their perceptions of the service recovery performance. If positive disconfirmation occurs, i.e., if consumers’ perceptions of the service recovery performance are greater than their expectations, a paradox might emerge (secondary satisfaction becomes more substantial than their pre-failure satisfaction). In contrast, there is a double negative effect in the case of negative disconfirmation as the service failure is followed by a flawed recovery (Smith and Bolton, 1998; McCollough et al., 2000). When customer satisfaction is negatively affected by a service failure, subsequent service recovery reactions may include negative WOM behavior (Hocutt et al., 2006). Positive evaluations occur when the recovery is understood as satisfactory (Matos et al., 2009). Satisfaction with service recovery is defined as positive customer evaluations of the service recovery experience (Bambauer-Sachse and Rabeson, 2015). The degree of success may depend on the type of service involved, the type of failure that occurred, and the type of recovery (Komunda and Osarenkhoe, 2012).

### Depicting Relationship Marketing Strategies

Over the past 20 years, there has been growing interest in the concept of relationship marketing among practitioners and academics (Morgan and Hunt, 1994; Sheth and Parvatiyar, 2000, 2002; Gummesson, 2002). Establishing a long-term relationship with customers generates an outstanding level of customer satisfaction, which, in turn, helps companies gain customers’ trust and loyalty and thus benefits the company overall (Valenzuela et al., 2010). Relationships help participants meet their objectives and depending on the stage of the connection (i.e., beginning stage, cultivating stage, or enhancing stage), the strategic implications might vary. As a result, firms must pay attention to different aspects at different stages, ranging from creating customer knowledge to shaping their perceptions. The relationship between an organization and its customers is strengthened by many marketing actions (Berry, 1983), which leads to further customer retention. Relationship marketing embodies tactical and strategic elements that can positively impact a firm (Lo and Campos, 2018). The implementation of relationship marketing endeavors to enhance the value of an enterprise to its customers and their long-term relationship (Bruhn, 2003). Relationship marketing further explores the marketing concept by focusing on the customer as an important representative figure and examining a promising way to acquire competitive advantages by exchanging information and becoming closer to the customer. This bond is genuinely advantageous to both parties. It allows both buyers and sellers to commit to achieving long-term benefits that offer greater chances for a successful relationship (Ganesan, 1994). Relationship marketing can be a challenging concept to implement, and the development of close bonds with customers is not always possible or alluring (Webster, 1992). Due to advances on the Internet and technology, sellers can utilize technology to process large amounts of data, derive specific information, and gain insight into customer preferences and behaviors. Therefore, companies can design more suitable solutions and products to meet their customers’ needs.

Relationship marketing refers to a customer’s perception of the extent to which the relationship fulfills his/her expectations, predictions, goals, and desires regarding the overall relationship (Wong and Sohal, 2002). A high degree of relationship marketing indicates that customers can rely on the service provider’s integrity and develop confidence in the service provider’s future performance because the past performance level has been consistently satisfactory (Tseng and Seidman, 2007). Kim et al. (2006) indicated that customers’ level of relationship marketing depends on various elements rather than only on the interpersonal relationship between service providers and their customers. Commitment, trust, and relationship satisfaction were considered as focal dimensions of relationship quality (Ulaga and Eggert, 2006; Verma et al., 2016). This study proposes that relationship marketing is composed of satisfaction and trust, while commitment belongs to consumer loyalty. Loyalty refers to a commitment to repurchase a product and/or service, and can be reflected by purchasing behaviors (e.g., frequency, intensity, and proposition) and WOM recommendations (Cossío-Silva et al., 2016).

Therefore, although service failures occasionally occur, service recovery can substantially impact relationship marketing because, through such marketing, customers gain confidence that long-term service providers will provide benefits. In contrast, when a customer is not satisfied with the service recovery measures taken by the enterprise. The relationship between the customer and the enterprise could be terminated (Zeithaml, 2002). Service recovery belongs to quality management, and its goal is to maintain a good relationship between enterprises and customers (Schweikhart et al., 1993). Good service recovery can improve customer satisfaction and promote customer trust in businesses, and compensatory service recovery can improve the degree of customer satisfaction (Yan and Jia, 2003; Wen, 2004).

Many scholars have shown that relationship marketing plays an intermediary role in the relationship between service recovery and consumer loyalty; thus, service recovery affects relationship marketing and consumer loyalty (Huang, 2006). Crosby et al. (1990) studied the relationship between service recovery and consumer loyalty in the insurance industry and confirmed the intermediary effect of relationship marketing (Chen and Fu, 2015). Huang (2006) used the banking industry as the investigation background and found that relationship marketing between customers and banks is affected by the perception of service recovery and plays an intermediary role in the relationship between service recovery and consumer loyalty. Huang (2006) also investigated the relationship between audio-visual rental customers and companies and further confirmed the intermediary role of relationship marketing. Therefore, based on the above analysis, this paper proposes the following hypotheses.

*Hypothesis 1*: Initiation positively influences relationship marketing.
*Hypothesis 2*: Response speed positively influences relationship marketing.
*Hypothesis 3*: Compensation positively influences relationship marketing.

Consumer loyalty is a desirable marketing indicator, and has been associated with positive organizational outcomes, such as sustainable competition (Kandampully et al., 2015; Huang et al., 2017; Rather et al., 2018; Affran et al., 2019; Rather and Hollebeek, 2019). Thus, a good service provider believes that a growth process usually involves customers, i.e., attracting new customers, retaining existing customers, and motivating customers to spend more and recommend products and services to other people. When customers experience a higher degree of relationship marketing with enterprises or service personnel, customer identification increases, customers are easily satisfied with the services provided, and customers’ willingness to find another service provider decreases (Crosby et al., 1990; Morgan and Hunt, 1994; Ruiz et al., 2008). Liu (2017) investigated the offline retail industry and found that adequately maintaining customer relationships promptly helps retail stores improve their customers’ sense of identity and cultivates dependence on the business. Rahman and Ramli (2016) conducted research that investigated the customers of some banking systems in northern Malaysia and verified the role of customer relationship marketing in the formation of consumer loyalty. In the Internet context, relationship marketing positively impacts consumers’ repeated purchase behavior (Jin et al., 2008; Zheng, 2008).

Relationship marketing includes customer satisfaction and customer trust, which have been shown to play a significant role in promoting consumer loyalty (Zhao, 2005). Specifically, Mayer et al. (2007) defined trust as the vulnerability of one party to the actions of another party based on expectations that the other party is performing in the desired way. The development of trust is considered a critical result of establishing long-term successful relationships among all parties involved. Moreover, many researchers have suggested that customers’ trust plays a significant role in building long-term relationships and achieving consumer loyalty (Feng and Zhang, 2009). For instance, research has shown that when customers consistently receive competent service, their trust levels increase, which results in their maintenance of long-term relationships with the firm (Balaji, 2015).

Customer satisfaction is customers’ overall attitudes toward a product, service or experience after their purchase (Leninkumar, 2017). Existing studies suggest that satisfaction positively affected consumer loyalty (Ruyter and Wetzels, 2000; Deng et al., 2010). Although satisfaction may not always be a reason that customers remain loyal (Gerpott et al., 2001), satisfied customers are more loyal than unsatisfied ones. This study thus proposes the following hypothesis:

*Hypothesis 4:* Relationship marketing positively influences consumer loyalty.

### Impacts of Service Recovery on Consumer Loyalty

The evidence suggests that appropriate service remedies can reduce or even eliminate consumers’ complaints and promote consumers’ trust in enterprises, a common marketing strategy of enterprises (Hart et al., 1990). Like offline service recovery, online service recovery includes responsiveness to customer complaints, resolving the communication process, and economic compensation for service losses (Parasuraman et al., 2005). Online companies are increasingly focused on reducing service failures, enhancing online service quality, and increasing customer satisfaction to attract more customers to make purchases and earn more profit (Zhao et al., 2014). Kelley et al. (1993) identified the top seven recovery strategies used to retain customers: discounts, correction, management/employee intervention, correction plus, replacement, apology, and refund. Smith et al. (1999) concluded that customers prefer recovery in both the value and form of recovery that matches the failure they experienced. Based on research investigating service failure and recovery (Smith et al., 1999; Mattila and Cranage, 2005; Weber and Sparks, 2009), apology and compensation are two key strategies used in service recovery. Moreover, the four attributes of perceived justice proposed by the extant research are compensation, response speed, apology, and recovery initiation (Chou, 2015). Based on research conducted by Smith et al. (1999), service recovery includes the four dimensions of compensation, response speed, apology, and initiation. Compensation, response speed, and apology are often in the business press, are particularly salient to customers, can easily be acted on by managers, and can be manipulated through written scenarios in an experimental context (Hart et al., 1990). Recovery initiation has received much attention in the business literature but has not been addressed empirically. We also expect interaction effects between the failure context and recovery attributes as subsequently described. According to the context of online takeout services, we selected compensation, response speed, and initiation as the three attributes of service recovery. Service recovery denotes a series of activities performed by companies to respond to a customer complaint regarding a service failure (Zhao et al., 2014). These strategies and activities aim to remedy problems with services and products (Kelley et al., 1993).

The most common definition of consumer loyalty was provided by Oliver (2010), who states that such loyalty occurs consistently in the future, thereby causing repetitive same-brand or same-brand-set purchasing despite any situational factors. Various authors have found that an increase in consumer loyalty increases profits, reduces the costs of acquiring new customers, and decreases the costs of serving current customers (Reichheld and Sasser, 1990). Increasing competition, particularly in the service industry, has caused firms to become very concerned with attracting potential customers and maintaining long-term relationships with their current customers, which is the aim of consumer loyalty (Bojei and Alwie, 2010). The measurement of consumer loyalty includes the following two aspects: attitudinal loyalty and loyalty in behavioral intention (Su et al., 2010; Zhang et al., 2010). Attitudinal loyalty is reflected in the willingness of consumers to recommend a service provider to other consumers or the commitment to re-patronize a preferred service provider (Chou, 2015). Behavioral intention is reflected in the frequency with which a customer chooses the same product or service compared to the total frequency with which this specific product or service is consumed (Chou, 2015).

In the online service context, the quality-of-service remedy has a positive impact on loyalty as expressed through customer behavioral intention. Etzel and Silverman (1981) show that service remedy positively affects loyalty through both customer attitude and behavioral intention. Further studies have shown that the response speed of service remediation, tangible compensation, and the initiation of remediation can promote the occurrence of repurchasing behavior and good WOM publicity intentions after the occurrence of service failures (Wang, 2014).

Service recovery is positively related to consumer loyalty, while service failure negatively relates to customer loyalty (Wang et al., 2011; Balaji et al., 2017). When loyal customers face service failure, they are more likely to re-evaluate their experience and service quality and then modify their decisions when competition is high (Fox et al., 2018). Therefore, more service providers have used service recovery as a means to improve customer retention (Vázquez-Casielles et al., 2012). Buttle and Burton (2002) asserted that most customers whose problems are resolved would repurchase, when organizations use an appropriate service recovery strategy. Recovery strategies thus aim to offset the dissatisfaction caused by service failure and reinforce positive WOM (Spreng et al., 1995). For example, travelers would recommend their airlines to others with service recovery efforts and/or incentives (Steyn et al., 2011). Additional evidence indicates that well-executed service recovery efforts can enhance customer satisfaction and loyalty (Buttle and Burton, 2002; Mostert et al., 2009; Steyn et al., 2011; Hu et al., 2013). Effective service recovery can increase customers’ trust, enhance consumer loyalty and increase customers’ willingness to repurchase in the future (Hu et al., 2013). Moreover, suppose merchants take effective service remedy measures. In that case, customers’ repurchase behavior increases, and customer satisfaction is significantly affected by the types of service remedy measures (oral apology, economic compensation, initiation, and response speed) (Zhang, 2013).

Based on the above reasoning, this study proposes the following hypotheses.

*Hypothesis 5*: Initiation has a positive impact on consumer loyalty.
*Hypothesis 6*: Response speed has a positive impact on consumer loyalty.
*Hypothesis 7*: Compensation has a positive impact on consumer loyalty.

**FIGURE 2:**
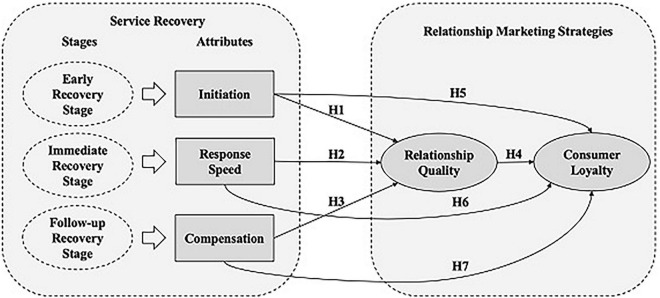
The proposed conceptual stage model of service recovery. H, hypothesis.

The revised service recovery cycle model elaborated in Figure 1, this study proposes the conceptual stage model of service recovery (Figure 2), which not only includes the three stages of service recovery and their recovery attributes in these stages. Notably, it examines how these recovery attributes influence relationship quality and customer loyalty.

## Research Methods

### Data Collection

The online survey was launched in March 2019. Our sample was purchased from a contracted marketing agency, who emailed our survey link to their participants. The participants in our survey were individuals who (1) had purchased via online food delivery apps, and (2) had reviewed and commented on service providers in the past 3 months (Table 1). When participants clicked on the survey link, they first accessed a page that described the study purpose, confidentiality, and privacy protocols. According to our contract with the marketing agency, we eliminated nine incomplete questionnaires and led to our final sample size of 301. Our response rate was 97.1%.

**TABLE 1:** Participants’ profile.

	*N*	%
**Gender (*n* = 301)**		
Male	149	49.50
Female	152	50.50
**Age (*n* = 301)**		
18–25 years old	100	33.22
26–35 years old	84	27.91
36–50 years old	93	30.90
50 years old and above	24	7.97
Mean (in years)	31.89	
Standard Deviation	7.89	
**Education level (*n* = 329)**		
High school and below	61	20.27
Bachelor’s college or below	86	28.57
Master’s degree	108	35.88
Ph.D. or doctorate degree	46	15.28
**Annual income level (*n* = 301)**		
50 thousand below	77	25.58
5–10 million	82	27.24
10–20 million	86	28.57
More than 200 thousand	56	18.60
Mean		11.48
Standard deviation		9.38

All items in the online survey instrument used the five-point Likert scale measurement method (1 = strongly disagree to 5 = strongly agree). First, we modified and developed the service recovery scale based on Mattila (2001); Mattila and Cranage (2005), Fan and Du (2006), and Ma et al. (2009), including nine items in the three attributes of initiation, response speed, and compensation. In Table 2, our factor analysis results showed that the internal consistency coefficients of initiation, response speed and compensation are respectively 0.769, 0.896, and 0.790. The Kaiser–Meyer–Olkin (KMO) value is 0.877. Second, relationship marketing in our study had two dimensions of customer satisfaction and customer trust. We developed the customer satisfaction scale based on Oliver (1980) and Leuthesser et al. (2003), which included a total of four items. We used Morgan and Hunt (1994)’s scale of customer trust with a total of four items. As shown in Table 3, the internal consistency coefficients of the customer satisfaction and customer trust scales are respectively 0.888 and 0.887. The KMO value of the scale is 0.831. Third, we examined consumer loyalty from attitudinal loyalty and behavioral intention (Table 4), according to Zeithaml et al. (1996) and Zhou (2005) using four items. The internal consistency coefficients of the attitudinal loyalty and behavioral intention scales are respectively 0.814 and 0.778. The KMO value of the scale is 0.822. In Table 5, the correlation coefficients among the three factors ranged from 0.26 to 0.61 (*P* < 0.01). Our survey instrument overall has a good level of reliability and validity.

**TABLE 2:** Results of the confirmatory factor analysis on service recovery attributes.

Items	Factor loading	Error variances	Ave	Cronbach’s α	Bartlett sphericity test (Df)	KMO
**Initiation**			0.57	0.77	113.05***	.88
(1) After service failure, the takeout merchant actively contacted me.	0.80	0.41				
(2) The merchants offered compensation after service failure.	0.80	0.48				
(3) The takeout merchant apologized to me after the service failure.	0.73	0.45				
**Response Speed**			0.64	0.90		
(1) In the case of service failure, the takeout merchant provided a timely response.	0.77	0.44				
(2) After service failure, the takeout merchant addressed my problems on time.	0.61	0.38				
(3) The takeout merchants addressed my problems in a short time.	0.63	0.39				
**Compensation**			0.60	0.79		
(1) In response to service failure, the takeout merchant provided product or money compensation.	0.84	0.38				
(2) In response to service failure, the takeout merchant gave me a gift or discount.	0.85	0.48				
(3) In response to the service failure, the delivery merchant promised me that I could return and exchange the goods free of charge.	0.60	0.42				

*All factor loadings were significant at the 0.001 levels.*

***p < 0.001. Model fit indices: n = 301, χ^2^ = 589.863, df = 329, CFI = 0.981, GFI = 0.884, NFI = 0.906, and RMSEA = 0.0452. AVE, average variance extracted.

**TABLE 3:** Results of the confirmatory factor analysis on relationship quality and customer loyalty.

Items	Factor loading	Error variances	Ave	Cronbach’s α	Bartlett sphericity test (Df)	KMO
**Satisfaction**			.61	.89	313.75***	.83
(1) I think it is the right decision to choose this takeout business.	0.77	0.38				
(2) It makes me happy to order from this takeout shop.	0.66	0.34				
(3) I like the service provided by the takeout merchant.	0.82	0.42				
(4) At work, I often hide my true emotional feelings.	0.82	0.41				
**Trust**			0.61	0.89		
(1) I believe the information provided by the takeout merchant.	0.62	0.42				
(2) I believe that the takeout business is concerned with the interests of its customers.	0.71	0.40				
(3) I believe that the takeout merchant is honest with its customers.	0.83	0.42				
(4) The takeout business makes me feel very relieved.	0.87	0.44				
**Attitudinal loyalty**			0.59	0.81	303.88***	.82
(1) I am willing to make positive comments about the takeout merchant and its products and services.	0.93	0.46				
(2) I would like to recommend the takeout merchant to my family and friends.	0.73	0.41				
**Behavioral intention loyalty**			0.59	0.78		
(1) I will continue to visit this takeout merchant.	0.79	0.38				
(2) If I need similar products in the future, I will choose this takeout merchant first.	0.88	0.42				

*All factor loadings were significant at the 0.001 levels.*

***p < 0.001. Model fit indices: n = 301, χ^2^ = 589.863, df = 329, CFI = 0.981, GFI = 0.884, NFI = 0.906, and RMSEA = 0.0452. AVE, average variance extracted.

**TABLE 4:** Descriptive statistics about respondents’ perceived service recovery, relationship quality and customer loyalty.

Items	*M*	*SD*
**Service recovery attributes**		
*Initiation* (*α* = 0.77)	3.83	0.83
(1) After service failure, the takeout merchant actively contacted me.	3.88	0.80
(2) The merchants offered compensation after service failure.	3.83	0.86
(3) The takeout merchant apologized to me after the service failure.	3.78	0.83
*Response speed* (*α* = 0.90)	3.85	0.85
(1) In the case of service failure, the takeout merchant provided a timely response.	3.84	0.83
(2) After service failure, the takeout merchant addressed my problems on time.	3.83	0.78
(3) The takeout merchants addressed my problems in a short time.	3.88	0.85
*Compensation* (*α* = 0.79)	3.86	0.88
1. In response to service failure, the takeout merchant provided product or money compensation.	3.91	0.67
2. In response to service failure, the takeout merchant gave me a gift or discount.	3.88	0.70
3. In response to the service failure, the delivery merchant promised me that I could return and exchange the goods free of charge.	3.81	0.77
**Relationship quality**		
*Satisfaction* (α = 0.89)	3.67	0.89
(1) I think it is the right decision to choose this takeout business.	3.71	0.86
(2) It makes me happy to order from this takeout shop.	3.70	0.82
(3) I like the service provided by the takeout merchant.	3.67	0.90
(4) At work, I often hide my true emotional feelings.	3.65	0.91
*Trust* (α = 0.89)	3.67	0.89
(1) I believe the information provided by the takeout merchant.	3.71	0.86
(2) I believe that the takeout business is concerned with the interests of its customers.	3.70	0.82
(3) I believe that the takeout merchant is honest with its customers.	3.67	0.90
(4) The takeout business makes me feel very relieved.	3.65	0.91
**Customer loyalty**		
*Attitudinal loyalty* (α = 0.81)	3.66	0.88
(1) I am willing to make positive comments about the takeout merchant and its products and services.	3.70	0.87
(2) I would like to recommend the takeout merchant to my family and friends.	3.64	0.88
*Behavioral intention loyalty* (α = 0.78)	3.66	0.85
(1) I will continue to visit this takeout merchant.	3.67	0.91
(2) If I need similar products in the future, I will choose this takeout merchant first.	3.65	0.86

**TABLE 5:** Correlations between variables.

	Initiation	Response speed	Compensation	Satisfaction	Trust	Attitudinal loyalty	Behavioral intention loyalty
Initiation	1.00						
Response speed	0.77**	1.00					
Compensation	0.69**	0.70**	1.00				
Satisfaction	0.76**	0.81**	0.78**	1.00			
Trust	0.67**	0.72**	0.80**	0.76**	1.00		
Attitudinal loyalty	0.32**	0.41**	0.35**	0.39**	0.56**	1.00	
Behavioral intention loyalty	0.64**	0.68**	0.77**	0.81**	0.77**	0.41**	1.00

*All factor loadings were significant at the 0.001 levels.*

**p < 0.01.

### Data Analysis

The data analysis in this study was divided into five steps. First, the descriptive statistics were analyzed to determine the characteristics and distribution of the measured variables, and the reliability of the scale was examined. Second, a measurement model with all dimensions was established by using confirmatory factor analysis (CFA) to test the fit of the measures. In the next step, a baseline path model was developed with structural equation modeling (SEM) to test H1 to H5. SEM was used to test the relationship between the independent variable and the dependent variable. For mediation to occur, the following four criteria must be met: (1) the independent variable should be significantly associated with the dependent variable; (2) the independent variable should be related to the mediator; (3) the mediator should be related to the dependent variable; and (4) the association between the independent and dependent variables must be reduced when the mediator is partially omitted. All variables (initiation, response speed, and compensation) were included in the measurement model (Figure 1). Fourth, the goodness of fit was tested under SEM to explore paths of influence and the magnitude of explained dependent variable. Finally, a structural model was developed, and the regression weights were compared to test the hypotheses.

## Results

### Respondents’ Sociodemographic Profiles

Around half of the participants (49.5%) were female, and 50.5% were male (Table 1). The mean age of the respondents was 31.9 years (*SD* = 7.9). Most of the respondents (35.9%) had a Bachelor’s degree or below, and the rest had a Master’s degree (28.6), a doctoral degree (15.3%), or high school and below (20.3%). The mean annual income of the respondents was $11,480 (*SD* = 9.49). Approximately one-quarter (25.6%) of the respondents had an annual income of $7,200 or below, 27.2% had an income between $7,200 and $14,400, 28.6% had an income between $14,400 and $28,800, and 18.6% had an income above $28,800.

### Descriptive Statistics and Correlation Analysis of the Variables

The results show that in service recovery, consumers have the highest positive perception of compensation (*M* = 3.86, *SD* = 0.88), followed by timeliness (*M* = 3.85, *SD* = 0.85) and loyalty (*M* = 3.83, *SD* = 0.83; Table 4). In general, consumers agree with service recovery overall. Regarding relationship marketing, consumers have the same perception of satisfaction and trust (*M* = 3.67, *SD* = 0.89); in consumer loyalty, consumers’ attitudinal loyalty (*M* = 3.66, *SD* = 0.87) and behavioral intention loyalty (*M* = 3.66, *SD* = 0.85) are also similar. Here, 1 indicates initiation, 2 indicates response speed, 3 indicates compensation, 4 indicates satisfaction, 5 indicates trust, 6 indicates attitudinal loyalty, and 7 indicates behavioral intention.

### Measurement Model

In the second step, the fits of the measures were assessed by using CFA. In this research, the model fits were evaluated through the comparative fit index (CFI), the goodness-of-fit index (GFI), the normed fit index (NFI), and the root mean square error of approximation (RMSEA). According to Bollen (1989) and Byrne (1998), a model is regarded as acceptable if the CFI exceeds 0.93, the NFI and the GFI exceed 0.90, and the RMSEA is less than 0.80.

Our CFA results show that the initial measurement model, which consists of nine items for three factors (i.e., initiation, response speed, and compensation), eight items for one factor (i.e., relationship quality), and four items for one factor (i.e., consumer loyalty), had acceptable fit indices (*n* = 301, χ^2^ = 594.995, DF = 344, CFI = 0.968, GFI = 0.875, NFI = 0.927, RMSEA = 0.047).

As all regression weights were significant (*p* < 0.01), the measurement model was further refined, as standardized residuals greater than 2.57 are considered to be statistically significant (Bollen, 1989), and large modification indices (those greater than 3.84) are considered to be statistically significant (Hayes, 2018). Furthermore, the discriminant validity of the measures was assessed by comparing the squares of the correlations between each pair of factors with their average variance extracted values (AVEs). The correlation coefficients among the three factors ranged from 0.26 to 0.61. Since the AVEs for all seven latent factors were higher than the squares of all correlation coefficients, the discriminant validity was acceptable. As shown in Tables 2, 3, the composite reliability values for initiation, response speed, compensation, satisfaction, trust, attitudinal loyalty and behavioral intention loyalty were 0.81, 0.90, 0.83, 0.90, 0.89, 0.89, 0.81, and 0.81, respectively. All values were higher than the suggested value of 0.80. The AVEs for all seven factors were equal to (for employee characteristics) or higher than the suggested value of 0.50. Thus, the convergent validity of the scale is acceptable.

### Baseline Model

**FIGURE 3:**
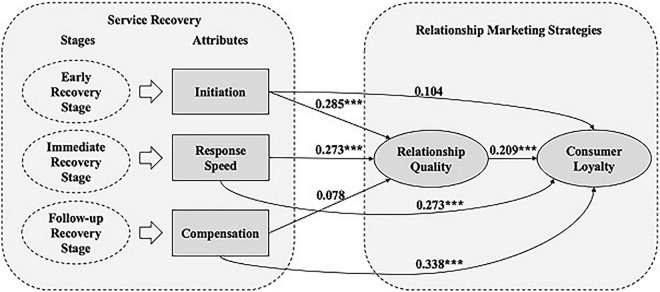
Results of structural modeling. *p < 0.05, **p < 0.01, and ***p < 0.001. *n* = 301, *_χ_^2^* = 589.863, df = 329, CFI = 0.981, GFI = 0.884, NFI = 0.096, and RMSEA = 0.0452.

In the next step, H1–H5 were tested by establishing a baseline structural model (*n* = 301) that included the initiation, response speed and compensation of service recovery as an exogenous variable and relationship marketing and service loyalty as endogenous variables. As shown in Table 6 and Figure 3, the baseline structural model has acceptable fit indices (*n* = 301, χ2 = 594.995, DF = 344, CFI = 0.968, GFI = 0.875, NFI = 0.927, RMSEA = 0.047). The SEM results reveal that response speed had a significant direct effect on relationship quality (β = 0.27; *p* < 0.001) and customer loyalty (β = 0.23; *p* < 0.001); therefore, H2 and H6 are supported. Compensation had a significant direct effect on customer loyalty (β = 0.34; *p* < 0.001), which supports H7, and initiation had a significant direct effect on relationship quality (β = 0.29; *p* < 0.001) and customer loyalty (β = 0.23; *p* < 0.001), which supports H6 and H2. Relationship quality had a significant direct effect on customer loyalty (β = 0.21; *p* < 0.001), which supports H4. However, compensation had no significant direct effect on relationship quality (β = 0.08). These findings thus support H1, H2, H4, H6 and H7.

**TABLE 6:** Results of SEM.

Effects	Direct effects	Indirect effects	Total effects
*H1*: Initiation → Relationship Quality	0.285***		0.29
*H2*: Response Speed → Relationship Quality	0.273***		0.27
*H3*: Compensation → Relationship Quality	0.078		0.08
*H4*: Relationship Quality → Customer Loyalty	0.209***		0.21
*H5*: Initiation → Customer Loyalty	0.104	0.02	0.23
*H6*: Response Speed → Customer Loyalty	0.273***	0.09	0.32
*H7*: Compensation → Customer Loyalty	0.338***	0.10	0.44

***p < 0.001.

*Model fit indices: n = 301, χ2 = 589.863, df = 329, CFI = 0.981, GFI = 0.884, NFI = 0.906, and RMSEA = 0.0452.*

## Conclusion

Our study results showed that response speed and compensation can help service providers increase customer satisfaction, maintain positive customer relationships and thus enhance customer loyalty. Although our results suggested that initiating service recovery does not significantly influence relationship marketing, it is a good indicator that service providers have realized the importance of service remediation and recovery by accounting for online reviews. It is impossible to recover customer trust with one-time service remediation after service failure, but customers have indicated their willingness to accept reasonable compensations or incentives for the purpose of maintaining customer loyalty.

Given the significant impacts that response speed has on consumer loyalty, it is essential to take response speed into account for online service providers by communicating with their customers in a timely manner. A responsive service provider should respond to positive reviews that encourages customer interactions, as well address negative reviews and comments, including dealing with customer frustration that should be managed with care, tact and diplomacy (Michel and Stefan, 2001). It is particularly important for online service providers to maintain effective communication with their customers by responding to messages, comments, and reviews on social media and other platforms (Bozkurt and Gligor, 2021). It has become an advantage of increasing customer loyalty by successfully tackling service failure (Mahmoud et al., 2018). For example, angry customers who had negative reviews may require a higher level of service recovery efforts, such as sending gifts with an apology card, offering free service. These active strategies can help service providers establish positive relationships with their consumers and improve customer loyalty.

Our study results have the following theoretical contributions. Our study focuses on the internal mechanism of the relationship between service recovery and consumer loyalty in the online service context, but also reaffirms the rationality of the path from service recovery to relationship marketing to consumer loyalty by exploring the prominent role of service recovery as a key event in long-term relationships with customers. Our results have also verified the importance of relationship marketing in the online service context, showed the positive impact of good relationship marketing on consumer loyalty, and contributed to the literature of customer relationship management.

In addition, our study results provide valuable practical implications. First, our study shows that service providers can use appropriate service recovery strategies to improve customer relationship and their loyalty with their services and/or brands. Unhappy consumers may be turned into loyal fans that leads to long-term profits and positive WOM for the service and brand. Response speed and compensation have been shown significant impacts on consumer loyalty. The primary goal of consumer complaints to merchants is to recover their loss; thus, response speed and compensation are essential factors of service remedy and key to whether consumers will consider repurchasing the merchant’s products.

Secondly, merchants should determine the deficiencies in their service system based on service errors and thoroughly conduct an overhaul on their service process to improve service quality and reduce the number of service errors. For instance, through ensuring smooth communication channels with consumers, effectively addressing customer complaints promptly while collecting information regarding service errors, and facilitating the rapid adjustment of their service process. There are specific differences between online customer service and offline business skill requirements. Therefore, it is better to establish professional online customer service processing specialists, clarify the service recovery implementation procedures, and grant customer service personnel specific authority to address unexpected situations promptly to reduce customer dissatisfaction.

This research has several limitations and recommendations for future research. First, this research examines the mediating effect of relationship quality on the relationship between service recovery and customer loyalty for online takeout in the context of China; thus, its findings may not be generalizable to other countries. Accordingly, future research could examine the same research question in other contexts, such as Western culture. Second, the measurements of relationship quality are satisfaction and trust, which most scholars accept, although some scholars view commitment as a third variable of relationship quality. Future research may commit to exploring more deeply the mechanism of action between service recovery and customer loyalty. Moreover, in future research, we could add more dimensions to the research model, such as the service failure type and remediation expectations, whose results would allow merchants to take targeted measures for service recovery. In addition, more detailed and in-depth research on different customer groups can be conducted to provide more targeted help for the practical management of takeout merchants.

## Data Availability Statement

The original contributions presented in the study are included in the article/supplementary material, further inquiries can be directed to the corresponding author/s.

## Ethics Statement

The studies involving human participants were reviewed and approved by the Academic Committee of Zhejiang Gongshang University. The participants provided their written informed consent to participate in this study.

## Author Contributions

JG: conceptualization, methodology, and manuscript writing. LY: data collection, data analysis, and manuscript writing. XX and PL: manuscript editing. All authors have contributed to and approved the manuscript.

## Conflict of Interest

The authors declare that the research was conducted in the absence of any commercial or financial relationships that could be construed as a potential conflict of interest.

## Publisher’s Note

All claims expressed in this article are solely those of the authors and do not necessarily represent those of their affiliated organizations, or those of the publisher, the editors and the reviewers. Any product that may be evaluated in this article, or claim that may be made by its manufacturer, is not guaranteed or endorsed by the publisher.
